# Exposure and Stretching of the Spinal Nerves

**Published:** 1872-12

**Authors:** 

**Affiliations:** Munich, Bavaria


ARTICLE II.—*Exposure and Stretching of the Spinal Nerves.* By PROF. VON NUSSBAUM, of Munich, Bavaria. (Translated and abridged by PROF. J. W. FREER, Chicago.)

The above title is applied by Prof. Von Nussbaum to a new surgical operation recently performed by him, for the cure of certain affections of the spinal nervous system, the nature of which will be made manifest in the following translation from his report:

"Rudolf Hailer, 23 years old, was, on the 1st of September, 1870, (during battle near Bazeilles,) severely struck and injured by means of the butt of a gun. The regions involved were the back of the neck, the left shoulder and elbow. Subsequently an abscess formed in the neck, which soon opened and healed kindly, but directly thereafter there appeared a condition of tonic contraction of the muscles of the left breast, arm, forearm and hand. It was not organic contraction, but distinctly a cramp, that increased in proportion to applied opposing forces. Anæsthesia was gradually developed on the back of the forearm, that finally reached a degree that would permit of deep cuts, without evidence of pain. Many skillful surgeons had already exercised their art, but the cramps continued the same, or at times increased, many times the fingers bored for hours with such force in the palm of the hand, as to cause the skin to be lacerated. The pectoralis major felt of the hardness of stone. Iodine, mercury, vesicants, gymnastics, galvanism, all were fruitlessly applied. Anæsthesia from chloroform caused complete relaxation only during its profoundest influence, at which times the arm was frequently confined to a splint, but long before the cessation of the narcosis, the cramps would return, and with such force that safety required the liberation of the limb.

"Also tenotomy was performed on many of the involved muscles with only transient relief.

"So had everything earthly been attempted, but poor Hailer remained the same unfortunate creature. Daily he begged to be freed from his unendurable condition, saying that he was ready to submit to any operation, even dislocation at the shoulder.

"I held Hailer's condition to be due to excessive irritability of the motor nerves, the spinal cord participating.

"Partly as a means of proof of my own diagnosis, and partly for the sake of advice as to a means of cure, I begged our much-respected physiologist, Prof. Voit, to examine the case.

"Prof. Voit held the lesion to be preponderatingly central, as appears in the following note:

"'The contraction does not depend upon blows received upon the arm, but rather upon those inflicted on the back of the neck. The cause cannot be peripheral, since the nerves of sensibility are paralyzed, while the motor are irritated, a separation of phenomena that obviously cannot obtain from causes of an eccentric character, because of the mingled relation of the two varieties of nerves.

"'The injured part is to be sought near the spinal cord, corresponding to the origin of the bronchial plexus, and involving the inferior cervical vertebra. It is supposable that the blow has not injured the cord, but rather the left roots, the posterior of which are quite separated, or at least sufficiently injured to interrupt their functions, while the anterior are not torn, but some condition has been induced which causes constant irritation, for example, a deviation of the vertebra, or a splinter of bone driven into the intervertebral foramina.

"'The lesion can also have its seat in the spinal cord itself, in which case the left posterior segment of the cord is involved, and not the posterior horn of gray matter, for if the latter was embraced in the change, there would be absence of sensibility in all parts beneath.

"'It is difficult to say if the sensibility will ever be restored, since possibly the posterior roots are completely separated.

"'The contraction must be relieved by removal of the cause, which is not well understood. Perhaps it is due to a contracting exudation, or to the pressure of a fragment of a neighboring vertebra. That the muscular cramp has continued so long without exhaustion, is a favorable sign.'

"Notwithstanding the refreshing character of the above instructions over a case that had already caused me much severe meditation, yet the views of Prof. Voit placed the hope of cure still more distant, for the spinal cord—whose participation in the pathology was almost certain—had as yet been considered as inaccessible to the surgeon's knife.

"Again I tried energetic doses of narcotics and the metals, since by the use of both one often sees useful results in central injuries of the nervous system, but in this instance the trouble only seemed to be enhanced thereby.

"My memory recalled several cases of epilepsy, that I, as pupil of the celebrated Romberg, had seen cured by the use of the knife. The removal of a sensitive cicatrix, or a compressed nerve, had frequently terminated fortunately. I re-read the beautiful experiments of Brown-Sequard upon guinea pigs, upon which artificial epilepsy was produced by injury of the ischiatic nerve; but alas, Hailer's case did not resemble epilepsy.

"In the winter session of 1860-61 of the children's clinic of Prof. Hanser's, I had operated upon a scrofulous child of six years, for anchylosis, and great angular deformity of the left elbow, by resection, and to the surprise of myself and my scholars we found that a long standing contraction of the third and fourth fingers was at once relieved through the incidental stretching of the ulnar nerve.

"This certainly very mild case resembled most nearly my present problem, and I determined with Hailer to lay bare all the implicated nerves, and to pull and stretch them, and since the spinal cord seemed to be implicated, I seized upon the plan to follow the four inferior cervical nerves to their points of exit from their respective foramina, and perhaps to relieve them from adhesions, and even to extend them.

"The patient was made aware of the uncertainty of the result, nevertheless, he acquiesced joyfully.

"On the 15th of February, the operation was performed, the patient being fully narcotized with chloroform. The first incision was in the track of the ulnar nerve, behind the inner condyle, the nerve elevated, gently stretched and replaced.

"A second incision was then made in the axilla, in the track of the axillary artery, and the surrounding nerves, singly drawn out of the wound, stretched, then replaced, the wound cleansed, and closed with sutures.

"Finally I made a cut four inches long in the direction of the largest curvature of the clavicle, as in operation for ligature of the subclavian art, divided the platysma myodes, exposed the plexus brachialis, lifted it out and followed each fasciculus with my right forefinger as far as their point of exit from the inter-vertebral foramina, a task not as difficult as I had apprehended.

"Then I pulled each in turn, backwards, forwards, upwards and downwards, in a word, disturbed them as near to the center as possible, and finally, performed moderate traction upon each, as if to draw them from their connection with the cord. During this latter encroachment upon the nerve centre, powerful contractions of the muscles of the corresponding side of the chest and arm were observable.

"The nerves, which seemed much longer than before the traction, were carefully replaced, and the wound closed.

"It is worthy of remark, that during these extensive manipulations, not a single abnormality was observed, no thickening, and no adhesions of the neurilemma. Only two subcutaneous vessels required ligatures. I deemed it unnecessary to interfere with the four superior cervical nerves, as there had been no symptoms implicating the phrenic, or any other division of this segment of nerves.

"The narcosis was protracted and the awakening correspondingly slow. With indescribable reflections, I awaited returning consciousness, that must decide whether the contractions had only been in abeyance during this state, as had been the case on all previous occasions. To my great joy and surprise these conditions did not reappear.

"The forearm and hand were again under voluntary control, and all the usual movements could be performed. Sensibility returned to the skin of the back of the arm, to such a degree that the slightest touch was perceived, with the eyes blindfolded.

"For a few days there was much pain in the wounds, especially in that of the neck, where my finger had followed the nerves.

"The suppuration from day to day became more and more abundant, to the extent of exciting apprehensions of pyemia, a condition then prevailing in the wards."

Prof. Nussbaum discourses as follows concerning pyemia:

"I know very well that many of my honored colleagues hold that pyemia is dependent upon *objective* conditions of wounds, and that the danger is not greater in hospital than in private practice. If this be true, how can one explain that in private practice this state seldom if ever occurs, while in hospitals, large numbers die of it yearly.

"Of the 11,000 operations that I have made, 4,000 were performed in the hospital clinic, and 7,000 in private practice, and in my two private hospitals, the latter surrounded by beautiful gardens and wholesome neighborhood. Of the 4,000 hospital patients, 200 died pyemic, while from the 7,000, only thirty were lost in this manner.

"To be sure, cases do arise in private practice, only it happens very seldom, and mostly where there are unavoidable cloacæ of offensive pus, while in the hospital wards the patients are often seized when the wounds are nearly healed.

"I acknowledge that the mechanical conditions of the wound, the constitutional disposition, and the local care bestowed, are very important factors, only the chief agent is the hospital air. Prof. Thiersch, of Leipsic, expressed the opinion, that if his clinical surgical patients could lie with their heads out of the windows, he would have no fear of pyemia.

"Since my declaration of so much distrust of hospital air it may be inferred that every protection was adopted, therefore he was caused to inspire through a sort of respirator containing lint, charged with carbolic acid, that the air might be purified from all low organisms.

"While I hold that such service cannot be denied to carbolic acid, still I do not belong to that class who cauterize fresh wounds and destroy good granulations, by its excessive use.

"It seems almost ludicrous for surgeons to besprinkle tables, knives, and other utensils with this agent, but those who recollect that ten years ago nearly all compound fractures, after eight to fourteen days, swam in stinking pus, then amputation, pyemia, and death, and who have since the introduction of Lister's method of bandaging, seen cases heal with a few drops of suppuration, must be blind not to see and acknowledge this as great advancement in sur ery."

We omit the remainder of the report concerning the vicissitudes that attended the healing process, which, as one might have predicted, was tedious.

The learned Professor concludes as follows:

"At the end of the eleventh week all was healed, therefore I could commence with the necessary gymnastic practices, which caused a rapid improvement in the movements of the limb, and in his general health, so rapid, that on the 102d day he was dismissed from the hospital, cured."

